# In Reply: Can Artificial Intelligence Make the Cut? Dissecting Large Language Model’s Surgical Exam Performance

**DOI:** 10.1016/j.mcpdig.2024.08.003

**Published:** 2024-08-13

**Authors:** Adam M. Ostrovsky, Joshua R. Chen, Vishal N. Shah, Babak Abai

**Affiliations:** Department of Surgery, Thomas Jefferson University, Philadelphia, PA

To the Editor:

We appreciate the thoughtful and comprehensive response to our paper, Performance of 5 prominent large language models in surgical knowledge evaluation: a comparative analysis.^1^ We are grateful for the opportunity to address the important points raised by the authors of the letter.^2^

### Reliability Concerns and Variability in Responses

We concur with the observation that the variability in responses from different large language models (LLMs) to identical queries poses a substantial reliability concern. As highlighted in our study, the inconsistency in responses across multiple trials indeed challenges the use of these models as dependable resources for medical education and clinical decision-making. Reliability, as is correctly pointed out, is paramount in health care, where inconsistent information can have serious repercussions.

Our findings reported that models like Doximity Generative Pretrained Transformer (Doximity GPT) and ChatGPT 4.0 reported higher reliability compared with others. However, even these models did not achieve perfect consistency across all trials. This variability underscores the necessity for users—students, educators, and professionals alike—to approach artificial intelligence (AI)-generated information with a degree of caution and to verify critical information through multiple sources.

Because our initial publication, OpenAI has followed up on the release of ChatGPT 4.0 with ChatGPT 4o (omni), which is billed as being faster, more accurate, and especially better at audio and visual understanding than previous models.^3^ Doximity has also released Doximity Answers, which is described as a tool capable of providing clinical answers with citations and sources.^4^ Although there are currently no studies assessing its reliability and accuracy, the ability to correctly and repeatedly provide references for answers would go a long way toward increasing trustworthiness and addressing the significant problem of AI hallucination, or, fabrications, by AI chatbots.^5^ New developments are continually emerging, and progress is being made in the reliability arena. Future studies can examine the effectiveness and accuracy of these tools in clinical settings, and their impact on health care outcomes and professional trust.

### Opacity of AI Systems and Trust

The issue of AI systems operating as black boxes is a valid and pressing concern. The complexity and lack of transparency in the internal workings of these models indeed make it challenging for users to understand the basis of the answers provided. This opacity can undermine trust, which is crucial for the integration of AI technologies in health care.

We agree that the lack of transparency necessitates rigorous oversight and the development of standards that ensure AI models are interpretable and their outputs understandable. Our study aims to highlight these issues and advocate for greater transparency and accountability in the deployment of AI in medical settings.

### Legal and Ethical Considerations

The ongoing legal scrutiny regarding the transparency and data usage of AI systems further emphasizes the need for comprehensive regulatory frameworks. The health care sector, given its sensitivity and the potential impact on patient outcomes, must adopt stringent standards to govern the use of AI technologies. This includes ensuring that AI models are trained on high-quality, unbiased datasets and that their decision-making processes are transparent and auditable. Our study is a step toward understanding the current capabilities and limitations of LLMs, but it is clear that more research is needed. Future studies should focus on improving the consistency and reliability of AI outputs, enhancing transparency, and developing regulatory frameworks that ensure these technologies can be safely and effectively integrated into medical practice.

In conclusion, although our study highlights the promising potential of LLMs in medical education, it also underscores the critical need for caution, ongoing assessment, and stringent regulation. We appreciate the engagement of the readers in this important dialog and hope that our collective efforts will contribute to the responsible and effective use of AI in health care.

## Potential Competing Interests

The authors report no competing interests.
